# Multi-Method Approach for Characterizing the Interaction between *Fusarium verticillioides* and *Bacillus thuringiensis* Subsp. *Kurstaki*

**DOI:** 10.1371/journal.pone.0092189

**Published:** 2014-04-16

**Authors:** Liliana O. Rocha, Sabina Moser. Tralamazza, Gabriela M. Reis, Leon Rabinovitch, Cynara B. Barbosa, Benedito Corrêa

**Affiliations:** 1 Department of Microbiology, Laboratory of Mycotoxins and Toxigenic Fungi, University of São Paulo, São Paulo, São Paulo, Brazil; 2 Department of Bacteriology, Laboratory of Bacterial Physiology, Oswaldo Cruz Institute, FIOCRUZ, Rio de Janeiro, Rio de Janeiro, Brazil; Soonchunhyang University, Republic of Korea

## Abstract

Bacterial antagonists used as biocontrol agents represent part of an integrated management program to reduce pesticides in the environment. *Bacillus thuringiensis* is considered a good alternative as a biocontrol agent for suppressing plant pathogens such as *Fusarium*. In this study, we used microscopy, flow cytometry, indirect immunofluorescence, and high performance liquid chromatography to determine the interaction between *B. thuringiensis* subsp. *kurstaki* LFB-FIOCRUZ (CCGB) 257 and *F. verticillioides* MRC 826, an important plant pathogen frequently associated with maize. *B. thuringiensis* showed a strong *in vitro* suppressive effect on *F. verticillioides* growth and inhibited fumonisin production. Flow cytometry analysis was found to be adequate for characterizing the fungal cell oscillations and death during these interactions. Further studies of the antagonistic effect of this isolate against other fungi and *in vivo* testing are necessary to determine the efficacy of *B. thuringiensis* subsp. *kurstaki* in controlling plant pathogens. This is the first report on the use of flow cytometry for quantifying living and apoptotic *F. verticillioides* cells and the *B. thuringiensis* Cry 1Ab toxin.

## Introduction

*Fusarium verticillioides* (Saccardo) Nirenberg ( = *F. moniliforme* Sheldon; teleomorph: *Gibberella moniliformis* Wineland) is an important plant pathogen commonly associated with maize, which is capable of causing seedling diseases and stalk and cob rots [1]. The ability to colonize plants endophytically makes it particularly difficult to control, leading to significant crop yield losses. The influence of abiotic and biotic factors, in addition to the morphological and genetic characteristics of maize, enables the conversion from an endophyte to a pathogen [2], [3]. This fungus also has the potential to produce several mycotoxins, primarily fumonisins, which are known to cause equine leukoencephalomalacia, pulmonary edema in swine, and cancer in rodents [4]. The consumption of fumonisin-contaminated grains is also associated with human esophageal cancer and neural tube defects in some regions of the world [5], [6].

Due to economic losses regarding *F. verticillioides* (*Fv*) contamination and the risks of fumonisin ingestion, many efforts have been made to control this fungus [7]. Resistant crop varieties, biological methods, and principally fungicides have been employed to manage *Fv* contamination; nevertheless, *Fusarium* strains exhibit a remarkable capacity to adapt and to become resistant to these strategies [8].

Pesticides have increased crop resistance for over four decades; nonetheless, the emerging, re-emerging, and endemic plant pathogens are still challenging crop safety worldwide [9], [10]. Moreover, chemicals may leave residues in grains, fruits, vegetables, and soil that may be harmful to the ecosystems and human health. The development of environmentally friendly crop-management practices for combating diseases represents a difficult task. The use of bacterial antagonists such as *Bacillus* [11]–[14], *Pseudomonas*, and *Streptomyces* [15], [16] as biocontrol agents (BCAs) is considered one of the most rational practices as part of an integrated management program to reduce pesticides in the environment [14], [17].

*Bacillus thuringiensis* (*Bt*) is a ubiquitous gram-positive spore-forming bacterium that produces parasporal crystals containing insecticidal proteins called crystal (Cry) or cytolytic (Cyt) toxins. This bacterium is found in a wide variety of habitats such as soils, dead and living insects, the plant phylloplane, and as an endophyte. The high abundance of *Bt* strains is attributed to its long-lasting spore viability [18], [19], ability to assimilate nutrients from diverse macromolecules [20], and its quorum sensing-regulated existence that can lead the bacterium from a virulent to a necrotrophic state [21]. *Bt* strains are classified according to the serological response differences in their flagellar antigens, and there are at least 71 serotypes and 84 subspecies [22], [23]. *B. thuringiensis* subsp. *kurstaki* (*Btk*), which produces lepidopteran-insecticidal Cry 1A proteins, including Cry 1Ab, is the most commonly employed strain used as a bioinsecticide against lepidopteran pest larvae [24].

Although many studies have focused on the activity of *B. thuringiensis* insecticidal proteins, the bacterium may also act as a potential BCA against a variety of plant pathogens due to its production of antimicrobial molecules, including zwittermicin A, chitinases, chitin-binding proteins, and quorum sensing-quenching enzymes [14], [20], [25], [26]. Together with chitinases, chitin-binding protein facilitates microbial attachment to fungal cell walls, which disrupts the cell polarity and leads to inhibition of cell growth. Previous studies have also demonstrated that *Bt* spore germination and vegetative cell growth were associated with the development of fungal hyphae in the soil. Furthermore, vegetative cells have been observed growing with the fungal mycelia, likely due to the ability of *Bacillus* species to cause the death of the fungus and thus survive the hyphal lysis products [27].

The aim of this study was to characterize the *in vitro* interaction of *F. verticillioides* and *B. thuringiensis* serovar *kurstaki* based on a multi-method approach. To our knowledge, this is the first report of the use of flow cytometry to quantify living and necrotic fungal cells and the Cry 1Ab toxin during the time-course of interaction between *Btk* and *Fv*.

## Materials and Methods

### Bacterial and fungal strains and their growth conditions

*F. verticillioides* MRC 826 [Programme on Mycotoxins and Experimental Carcinogenesis (PROMEC), Tygerberg, Republic of South Africa] was obtained from a culture growing on Spezieller Nährstoffarmer agar by monosporic isolation and was used in all experiments. This isolate is capable of producing high levels of fumonisins [28]. The fungal strain was inoculated onto V8 agar in Petri dishes and incubated under continuous fluorescent white light for 7 days at 25°C in a BOD incubator (Thermo Scientific, Wilmington, DE), after which the colony surface was gently scraped off and transferred to a tube containing 50 mL of sterile distilled water. The spores were counted with a hemocytometer, and the concentration was adjusted to 1×106 spores/mL [29].

*Bacillus thuringiensis* subsp. *kurstaki* LFB-FIOCRUZ (CCGB) 257, which produces the Cry 1A toxin group, was provided by Dr. Leon Rabinovitch from the Culture Collection of *Bacillus* and Related Genera (CCGB; Oswaldo Cruz Institute, Rio de Janeiro, Brazil). This *Btk* strain was isolated from moist soil from the Morretes Village, Paranaguá, Paraná State, Brazil prior to 1995. It was identified and characterized by Dr. Leon Rabinovitch and Dr. Tania V. Guaycurus at the Bacterial Physiology Laboratory in the Oswaldo Cruz Institute in collaboration with the Pasteur Institute, Paris, France. The LFB-FIOCRUZ (CCGB) 257 strain exhibits proteolytic and amylolytic properties, is mesophilic, and produces a bi-pyramidal crystal. The serovar *kurstaki* (H3a, 3b, 3c), which produces flagellar antigens of serotype “H3”, was characterized by the Pasteur Institute.

The cultures were stored lyophilized; bacterial spores and crystal biomass were obtained by inoculating *Btk* LFB-FIOCRUZ (CCGB) 257 into Erlenmeyer flasks containing 250 mL of Nutrient Broth (Difco Laboratories, Detroit, Mich.) and incubating at 30°C on a rotary shaker (100 rpm) for 72 h. The cultures were centrifuged at 12,000 rpm for 30 min at 4°C, and the cell pellets were stored at −20°C [30] until use. The protein concentration was determined with Bradford reagent (Sigma-Aldrich Corporation, St. Louis, MO), and measurements were made with a NanoDrop 2000 Spectrophotometer (Thermo Scientific, Wilmington, DE), using bovine serum albumin (BSA) as the standard [31]. A working solution of the *Btk* spore and crystal biomass was made by diluting to 10 µg/mL, which was the minimum inhibitory concentration for *Fv* growth under our laboratory conditions (data not shown).

About 50 µL of Btk biomass (10 µg/mL) was centrally inoculated onto potato dextrose agar (PDA) using a micropipette. After inoculum adsorption, 50 µL of *Fv* (1×106 spores/mL) was centrally inoculated onto each PDA Petri plate. PDA medium was chosen since it can provide for *Fusarium* species' growth, in addition to bacterial growth. The microscopy, immunofluorescence, and cytometry experiments were conducted with five replicates after 3, 5, and 7 days of incubation under continuous white-light illumination at 25°C. The radius measurements of the pure and mixed (*Fv*+*Btk*) cultures were recorded for 20 days for five replicates each, after which the presence of the fumonisins B_1_ and B_2_ was determined.

### Flow cytometry analysis for the quantification of fungal cells

Five replicates were analyzed after 3, 5, and 7 days of incubation. The cultures were removed from the agar by scraping, transferred into a 50-mL tube containing sterile distilled water and Tween 80 (2 drops/100 mL water), and centrifuged at 7000 rpm for 20 min. The cells were rinsed with the Tween 80/water solution twice and resuspended in 20 mL phosphate buffered saline (PBS), after which a 4 mL aliquot was filtered through a 70-µm mesh-sized cell strainer [Becton Dickinson and Company (BD), San Jose, CA] to obtain a uniform cell suspension. The cell solution was stained with 10 µL of 1 µg/mL Calcofluor White (CFW; Sigma-Aldrich, Buchs, Switzerland) at a final concentration of 0.05 µg/mL CFW and stained with 5 µL of 7-aminoactinomycin (7-AAD), utilizing the PE Annexin V Apoptosis Detection Kit (BD, San Jose, CA) per the manufacturer instructions. The cells were incubated with CFW and 7-AAD in the dark for 15 min and stored on ice until the analysis.

The living and necrotic fungal cells were quantified by flow cytometry performed using a FACS Canto II system (BD, San Jose, CA) equipped for CFW (λ_ex_, 365 nm; λ_em_, 430 nm), using violet laser excitation (405 nm) with detection in the Pacific Blue channel (405–450/50 nm), and 7-AAD (λ_ex_, 546 nm; λ_em_, 647 nm), using blue laser excitation (488 nm) with detection in the PerCP (peridinin chlorophyll A protein) channel (650 nm). A minimum of 30,000 events per sample were acquired; the data were collected using a linear representation for the side scatter (SSC) and forward scatter (FSC) and a logarithmic representation for the fluorescent signals. The analyses were performed using FlowJo software (Tree Star Inc., Ashland, OR). The necrotic fungal cell population was gated to separate it from the dead bacterial cell population. For verifying *Fv* autofluorescence and cross reaction between the *Btk* and CFW, bacterial cells stained with CFW and fungal cells not labeled with the fluorochrome were used as negative controls. For the 7-AAD negative controls, pure cultures of living bacterial and fungal cells were stained with 7-AAD. The negative controls were utilized to determine the background of each fluorescent marker. For the 7-AAD positive controls, the bacterial and fungal cells were incubated at 100°C for 30 min in a water bath [32],[33].

### Transmission electron microscopy (TEM)

After the incubation periods on PDA, 1-cm^2^ portions of agar containing interaction regions of both microorganisms and samples from pure fungal and bacterial cultures were fixed with 2% (v/v) glutaraldehyde in 0.1 M cacodylate buffer, pH 7.2, for 2 h at room temperature and then overnight at 4°C. The samples were rinsed with cacodylate buffer and fixed in 1% (w/v) osmium tetroxide before dehydration in a graded series of ethanol concentrations and embedment in Epon 812 resin. Ultrathin sections were collected on 200-mesh nickel grids coated with Formvar and stained with uranyl acetate and lead citrate. The grids were examined with a JEOL 1010 transmission electron microscope (JEOL, Tokyo). For each replicate, three ultrathin sections were examined [11].

### Scanning electron microscopy (SEM)

The pure cultures and treatments were placed in a 40% glutaraldehyde solution for 24 h, dried at 42°C for at least 48 h, and fixed to appropriate aluminum bases. The material was then sputtered with gold and examined with a Leo-440i-SEM scanning electron microscope (Leo electron microscopy, Cambridge, UK) [29].

### Indirect immunofluorescence assay

Pure and treated cultures of *Fv* and *Btk* were analyzed during the incubation period. The cells were removed by scraping, washed 3 times with PBS, and incubated for 30 min in PBS/BSA (1%) at room temperature before adding rabbit polyclonal antibody against *Bt* Cry 1Ab toxin (1∶1000; Abcam, Cambridge, MA); the incubation was for 30 min in the dark under gentle rotation (60 rpm). The cells were washed three times with PBS/BSA and incubated with a goat polyclonal secondary antibody to rabbit IgG coupled to Alexa Fluor 488 (1∶1000; Abcam, Cambridge, MA) under the same conditions. The cells were washed three times with PBS/BSA and 10 µL of CFW (1 µg/mL) was added. The cells were resuspended in 40 µL of a solution composed of glycerol (500 µL), PBS (400 µL) and n-propyl gallate (0.022 g) (Sigma-Aldrich, Buchs, Switzerland). Slides were prepared and analyzed with an EVOS FL Cell Imaging System (Advanced Microscopy Group, Life Technologies, Foster City, CA) using the DAPI (diamidino-2-phenylindole) channel for CFW and the GFP (green fluorescence protein) channel for Alexa Fluor 488 (λ_ex_, 495 nm; λ_em_, 519 nm). The background of each fluorescent marker was determined with negative controls of the *Btk*, *Fv*, and *Btk*+*Fv* treatments incubated with the secondary antibody, but not with the primary antibody. For the CFW controls, the bacterial cells were stained with the fluorochrome, while the fungal cells were not [34], [35].

### Flow cytometry for quantification of the Cry 1Ab toxins

The cells were treated as described above, but omitting the CFW staining. Quantification of the Cry 1Ab toxin was performed with the FACS Canto II (BD, San Jose, CA) equipped for Alexa Fluor 488 (λ_ex_, 495 nm; λ_em_, 519 nm) using blue laser excitation (488 nm) with detection in the FITC (fluorescein isothiocyanate) channel (530/30 nm). The collection and analysis were as previously described for the flow cytometry analysis of the fungal cells. For negative controls, the cells from all treatments were incubated only with the secondary antibody [33].

### Fumonisin analysis

*Fv* (1×10^6^ spores/mL) and *Fv* plus *Btk* biomass (10 µg/mL) were inoculated onto PDA (five replicates/treatment) and incubated at 25°C for 20 days [12]. Fumonisins were extracted from the PDA culture with 100 mL of methanol and water (3∶1, v/v) followed by shaking for 45 min. The samples were filtered through Whatman grade 4 (12 cm) filter paper and the pH was corrected to pH 5.8–6.5 with 1 N NaOH, if necessary. The fumonisins were purified by transferring 10 mL of the filtrate to a minicolumn containing 500 mg of ion-exchange silica (BondElut SAX-Varian, Palo Alto, CA, USA) previously conditioned with 5 mL methanol and 5 mL methanol/water (3∶1, v/v) at a flow rate of 1 mL/min. The fumonisins were eluted with 15 mL methanol/acetic acid (99∶1, v/v), maintaining the same flow rate. The product was evaporated and the residue was separated by high performance liquid chromatography (HPLC) and resuspended in 1 mL acetonitrile/water (50∶50, v/v). A 50-µL aliquot of the sample extract was diluted with 50 µL OPA reagent (40 mg ortho-phthalaldehyde dissolved in 1 mL methanol, diluted with 5 mL 0.1 M sodium tetraborate solution, and supplemented with 50 µL β-mercaptoethanol) and shaken for 30 seconds. Two minutes after the addition of the OPA reagent, the solution was injected into a Shimadzu LC-10AD liquid chromatograph equipped with a 20 µL fixed loop injector (Rheodyne, Rhonert Park, CA) [36], [37]. After separation on a C-18 reverse phase column (Phenomenex, Torrance, CA, 5 ODS-20, 150×4.6 mm), the fumonisins were detected with an RF-10AXL fluorescence detector (λ_ex_, 335 nm; λ_em_, 335 nm). An acetonitrile/water/acetic acid (96∶104∶1, v/v/v) solution was used for the mobile phase. Chromatography was conducted with column temperature of 30°C, maintained in an oven, and a flow rate of 1.0 mL/min,at room temperature (22–23°C). The retention times of the FB_1_ and FB_2_ under these conditions were 9 and 20 min, respectively. Calibration curves were utilized for quantifying the fumonisin with correlation coefficients of 0.9927 (FB_1_) and 0.9950 (FB_2_). The quantification limit was 0.015 µg/g for FB_1_ and FB_2_, and the mean recoveries from five replicates for each were 92.38% (SD, 13.68%) and 85.39% (SD, 6.87%) for FB_1_, and FB_2_, respectively.

### Statistical analyses

Statistical comparisons were performed using ANOVA and Tukey tests, as the data were normally distributed. The differences were considered significant when *p*-values were <0.05. The statistical tests were conducted using Assistat software [38].

## Results

### Accuracy of flow cytometry analysis for quantification of living and dead *Fv* cells and *Btk* Cry 1Ab toxin during the time course of interaction

**Figure 1:**
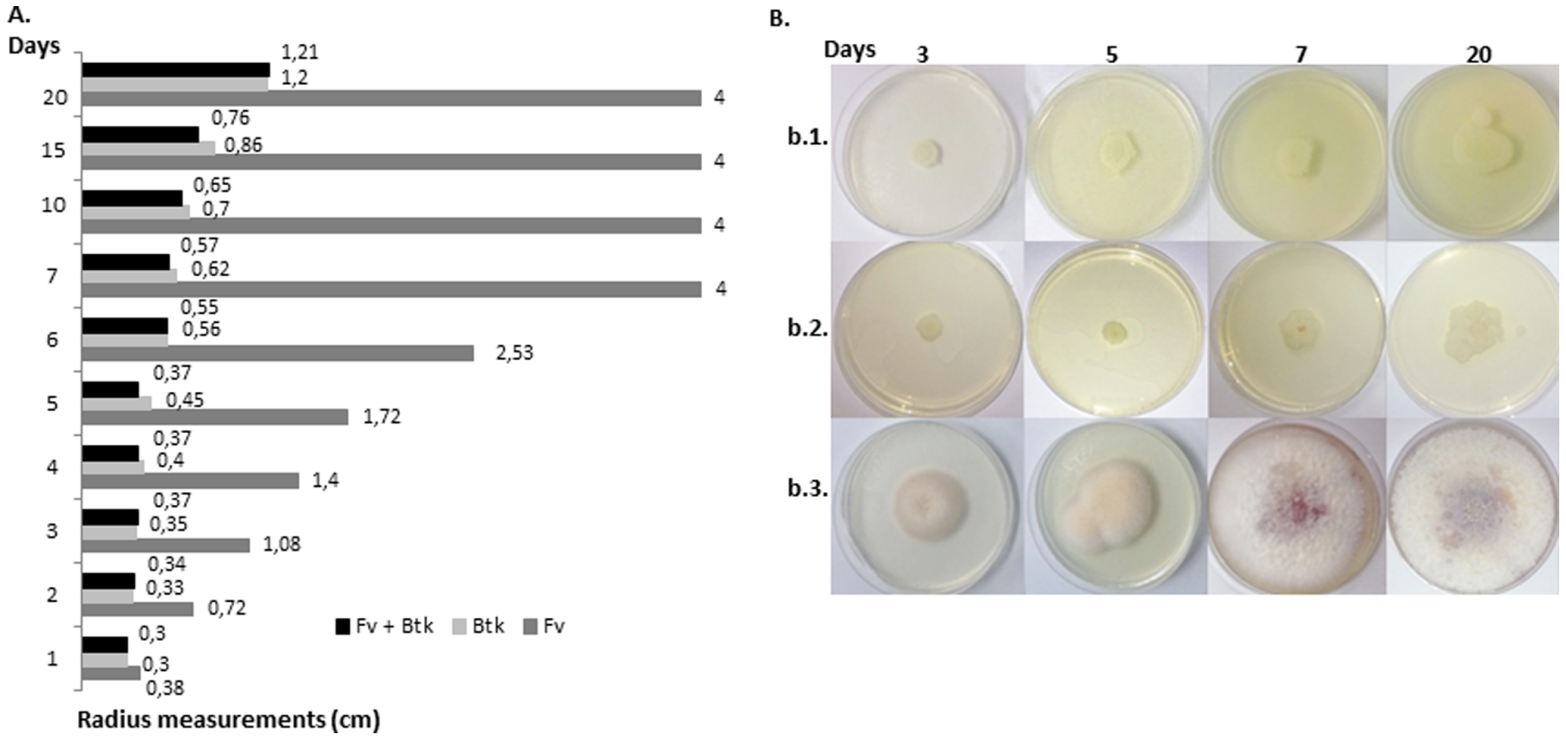
Effect of *Bacillus thuringiensis* subsp. *kurstaki* (*Btk*) on the mycelial growth of *Fusarium verticillioides* (*Fv*). (A) Each bar is the average of three radius measurements (cm) of pure fungal colonies and the interaction of *Fv* and *Btk* (five replicates) during 20 days of growth on potato dextrose agar. The differences were considered significant when *P* values were <0.05, according to ANOVA and Tukey tests. Although there were no significant differences between *Fv*+*Btk* and *Btk*, the average of the radius measurements were significantly reduced with *Fv*+*Btk* when compared to *Fv* culture, from the second to the 20th day of incubation.; (B) aspect of the colonies after 3, 5, 7 and 20 days: *Btk* (b.1); *Btk*+*Fv* (b.2); and *Fv* (b.3).

**Figure 2:**
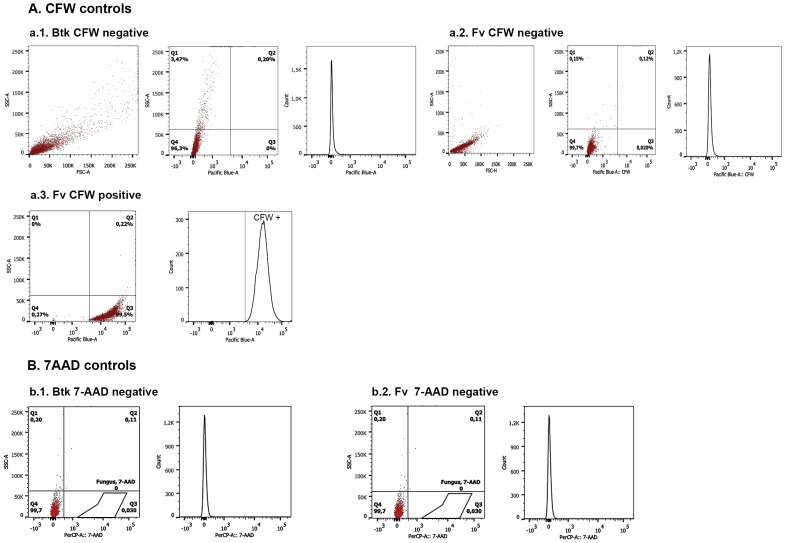
Flow cytometry analysis for *Bacillus thuringiensis* subsp. *kurstaki* (*Btk*) interacting with *Fusarium verticillioides* (*Fv*). Histograms show the number of cells versus the fluorescence intensity. Dot plot graphs show the cell size (SSC) versus the cellular complexity and the SSC versus the fluorescence intensity. The vertical lines define the baseline above which the fluorescence is positive. **Figure 2(A)** **.** Calcofluor White (CFW) controls and their respective histograms: negative, *Btk* stained with CFW (a.1); negative, *Fv* cells not stained with CFW (a.2); and positive, fungal cells stained with CFW (a.3). **Figure 2(B)** **.** 7-Aminoactinomycin (7-AAD) controls: negative, living *Btk* cells (b.1.); negative, living *Fv* cells (b.2.); positive, dead *Btk* cells (b.3.); and positive, dead *Fv* cells (b.4.). The fungal cells were gated based on the forward (FSC) and side scatter (SSC) and previous analyses with 7AAD. **Figure 2(C)** **.** Analysis of the *Fv* and *Fv*+*Btk* cells labeled with CFW and 7-AAD after 3 (c.1), 5 (c.2), and 7 (c.3) days.

To determine whether *Btk* LFB-FIOCRUZ (CCGB) 257 could be a potential BCA against *Fv*, flow cytometry tests were used to identify either apoptotic or living fungal cells. During the culture growth for the analyses, it was observed that *Fv* increased its biomass faster than did *Btk* on PDA medium, as demonstrated by the average of three radius measurements of the colonies alone versus those of interactions between *Fv* and *Btk* (five replicates) during the period of analysis (Figure 1). The use of CFW and 7-AAD fluorochromes was based on the ability of CFW to bind to the cell wall chitin of both the living and dead fungus. The 7-AAD permeates the membrane of late-apoptotic and dead cells, and binds to the DNA. Thus, it was possible to verify if the interaction led to fungal death or growth inhibition. Furthermore, through a gating strategy, it was possible to distinguish between the bacterial and fungal populations undergoing apoptosis (Figure 2).

Positive and negative controls were used to validate the flow cytometry conditions and to analyze the *Fv* and *Btk* interaction. This technique allowed for the quantification of live and dead fungal cells and the Cry 1Ab bacterial toxin in terms of percentages (Figure 2). *Btk* cells were stained with CFW with negligible background detected (Figure 2a.1). *Fv* cells were also stained with CFW, and almost 100% of the cells were counted (Figure 2a.3). Quadrants were defined based on positive and negative fungal populations and a negative bacterial population for CFW (Figure 2A). For the 7-AAD, living bacterial, fungal, and bacterial plus fungal cells were used as negative controls (Figure 2b.1 and b.2). Quadrants were established after analyzing both microorganisms and their interaction. Dead *Fv* and *Btk* cells were observed as distinct populations since it was possible to select the *Fv* population by gating (Figure 2b.3 and b.4). Not all of the bacterial cells were in an apoptotic stage after 30 min at 100°C, which can be explained by the presence of spores and crystals that are resistant to high temperatures and therefore not labeled by the 7-AAD (Figure 2b.3 and b.4).

**Figure 3:**
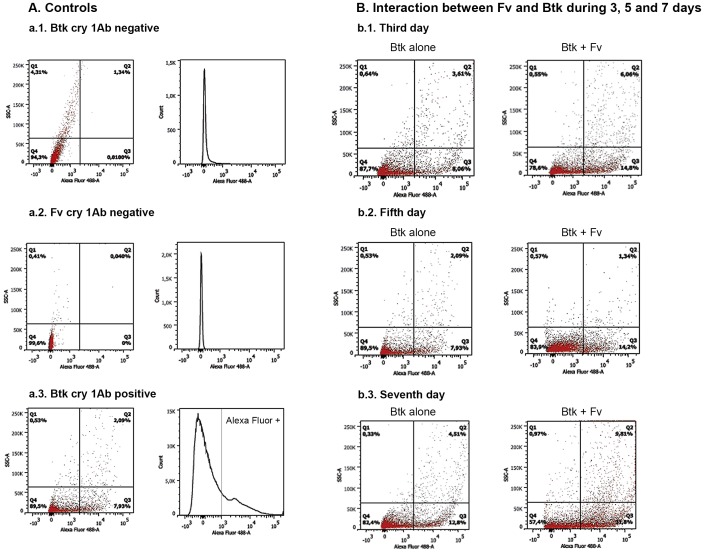
Flow cytometry analysis for quantification of the Cry 1Ab toxins. Histograms show the number of Cry 1Ab toxins versus the fluorescence intensity. Dot plot graphs show the protein size (SSC) versus the fluorescence intensity. The vertical lines define the baseline above which the fluorescence is positive. (A) Experimental controls: negative, *Btk* cell suspension treated with secondary antibody coupled with Alexa Fluor 488 (a.1); negative, *Fv* cells treated with the first and secondary antibodies (a.2); and positive, *Btk*+*Fv* cell suspension treated with the first and secondary antibodies (a.3). (B) Analysis of Cry 1Ab production after 3, 5, and 7 days of interaction between *Fv* and *Btk*: *Btk*+*Fv* interaction and *Btk* Cry 1Ab toxin stained with Alexa Fluor 488 on the third (b.1), fifth (b.2), and seventh (b.3) days of interaction.

Cry 1Ab was treated with an antitoxin antibody and stained with Alexa Fluor 488 in order to determine the crystal production, and consequently, the bacterial spore production during the analysis period. The quadrants were delimited based on the dot plot graphic of the negative control for the Cry 1 Ab toxin (Figure 3A).

### In vitro suppressive effect of *Btk* against *Fv*

**Figure 4:**
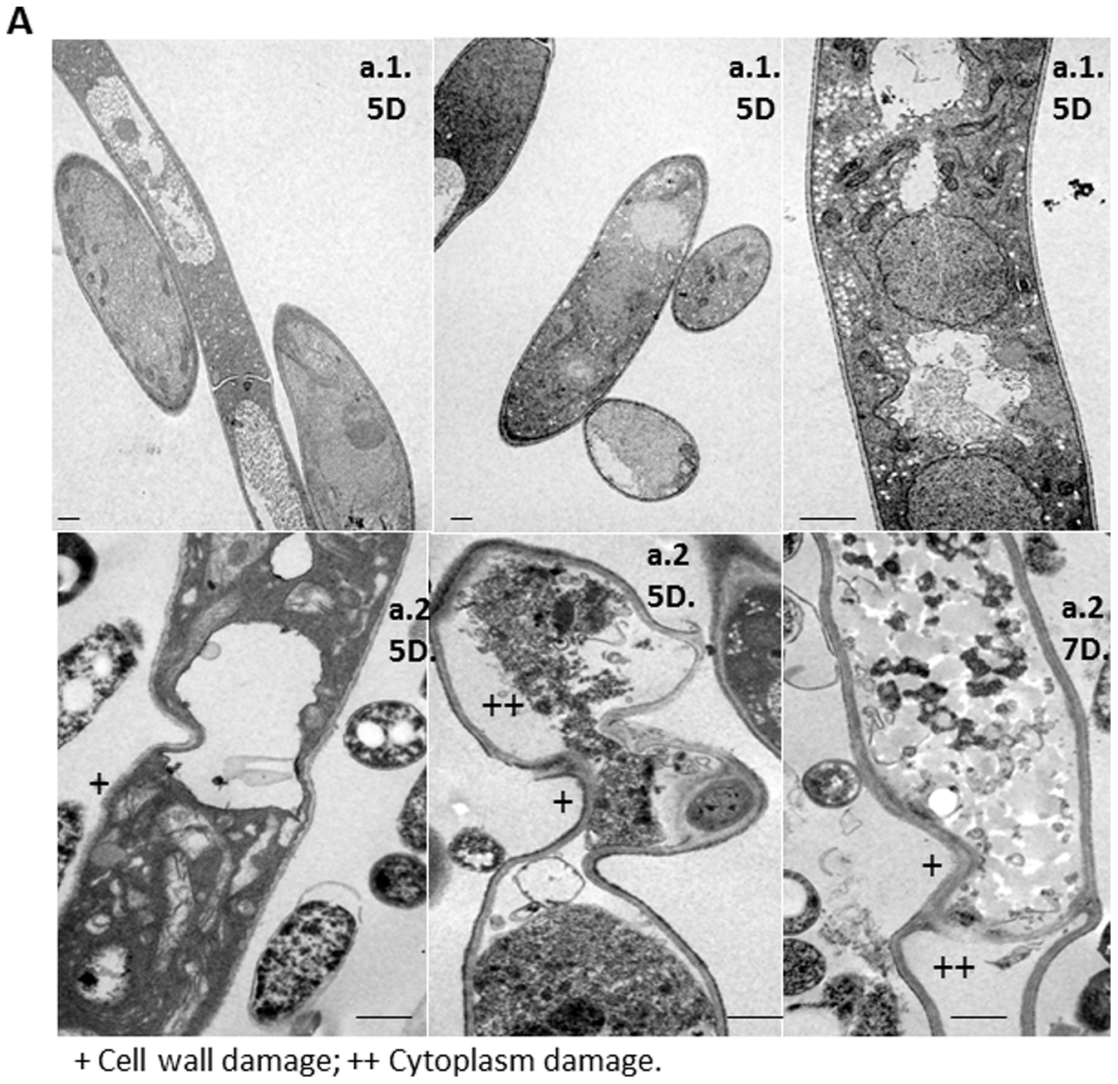
Microscopic observations of *Fusarium verticillioides* (*Fv*) in contact with *Bacillus thuringiensis* subsp. *kurstaki* (*Btk*). *Fv* and *Btk* were co-cultured on potato dextrose agar and microscopically examined after 5 (5D) and 7 (7D) days of growth. **Figure 4(A)** **.** Transmission electron microscopy (TEM): *Fv* cells from pure culture (a.1) and with damaged cell walls and disorganization of the cytoplasm – scale bar: 1 µm (a.2). **Figure 4(B)** **.** Scanning electron microscopy (SEM): *Fv* cells from pure culture – scale bars: 10 µm and 20 µm (b.1); *Btk* cells from pure culture – scale bar: 10 µm (b.2); intumescent hyphae and sparse fungal growth – scale bars: 5 µm, 10 µm and 20 µm (b.3); and *Btk* spores and crystals around the hyphae – scale bars: 5 µm and 10 µm (b.4).

The *Btk* LFB-FIOCRUZ (CCGB) 257 strain was found to show significant antagonistic activity against *Fv* from the second to the twentieth day, based on the radius measurements of the five replicates (Figure 1). After 3, 5, and 7 days of interaction, the colonies were viewed by transmission electron microscopy (TEM) and scanning electron microscopy (SEM), in addition to the indirect immunofluorescence and flow cytometry analyses. After 3 days of incubation, no marked changes were observed by TEM or SEM in the *Fusarium* cells (data not shown). On the fifth and seventh days, the *Fusarium* cells appeared damaged, as evidenced by cell-wall irregularities and by disorganization of the cytoplasm. In contrast, the *Fv* pure cultures showed intact hyphae and abundant microconidia formation when the colonies were analyzed by TEM (Figure 4A). In the SEM analysis, strong fungal cell wall thickening was seen in response to the interaction after 5 and 7 days of incubation; sparse fungal growth and numerous *Btk* cells and spores were also discernible. As a result of the interaction, high numbers of *Btk* spores and cells were visualized between the hyphae and microconidia; the *Btk* pure culture produced vegetative cells in profusion (Figure 4B).

Flow cytometry dot plot graphs demonstrated a significant reduction of the fungal cells from the third (10.93%) to the fifth day (4.19%; *p*<0.05), although the percentage of cells remained virtually the same from the fifth to the seventh day (3.48%). Interestingly, fungal cell death was observed on the seventh day of incubation (5.23%), in agreement with the TEM and SEM observations (Figure 2C). The fungal cultures alone showed irrelevant changes in the percentage of cells for the three periods analyzed.

**Figure 5:**
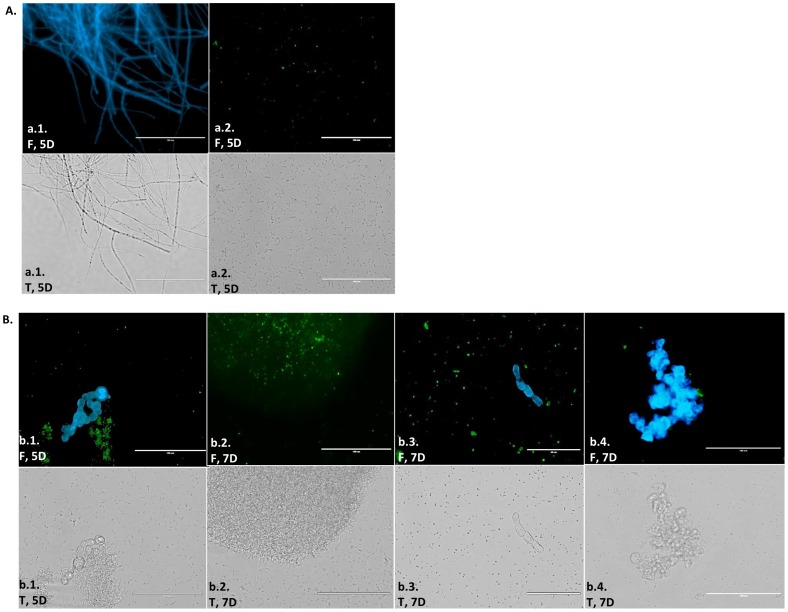
Interaction between *Fusarium verticillioides* (*Fv*) and *Bacillus thuringiensis* subsp. *kurstaki* (*Btk*) monitored by immunofluorescence microscopy. Cells were treated as described in the Materials and Methods. Fungal cells were stained with Calcofluor White (CFW; blue fluorescence) and crystals containing Cry 1Ab toxin were labeled with Alexa Fluor 488 (green fluorescence) after 3 (3D), 5 (5D) and 7 (7D) days of interaction. Fluorescence (F) and transmission (T) images: (A) Controls: *Fv* cells stained with CFW – scale bar: 100 µm (a.1) and *Btk* Cry 1Ab toxin labeled with Alexa Fluor 488 – scale bar: 100 µm (a.2); (B) interaction between *Btk* and *Fv*: intumescent hyphae and Cry 1Ab toxin, 5D – scale bar: 100 µm (b.1); Cry 1 Ab toxin, 7D – scale bar: 100 µm (b.2); and intumescent hyphae and Cry 1Ab toxin, 7D – scale bar: 50 µm (b.3 and b.4).

The immunofluorescence assay using CFW to stain the fungal cell walls allowed for the identification of many sectors of hyphal thickening on the fifth day, a characteristic that was remarkably noted on the seventh day (Figure 5B). On the third day, a few crystals were observed and the fungal cells were basically intact (data not shown), but by the fifth and seventh days, crystals containing Cry 1Ab toxin were mostly free and in abundance throughout the cultures (Figure 5B).

By the end of the experiment, the treated cultures were primarily colonized by the *Btk* LFB-FIOCRUZ (CCGB) 257 strain, demonstrating its relevance as a BCA. The morphological alterations observed by TEM, SEM, and the immunofluorescence assays subsequently confirmed the flow cytometry results. No such changes were noted in the control cultures.

### Effects of *Btk* Cry 1Ab production

The production of the Cry 1Ab toxin by the *Btk* LFB-FIOCRUZ (CCGB) 257 strain was analyzed after 3, 5, and 7 days of incubation by utilizing specific antibodies followed by analysis with flow cytometry. This experiment was conducted based on the observations of the indirect immunofluorescence assay, which was possible to visualize high quantity of the toxin and *Btk* spores all over the treated culture (Figure 5B). In *Btk* control cultures was observed 10.02%, 11.67% and 17.31% of Cry 1Ab toxins in 3, 5 and 7 days, respectively (Figure 3, B). A significant increase of Cry 1Ab toxins was verified in cultures with *Fv* added to the *Btk* LFB-FIOCRUZ (CCGB) 257 strain in comparison to the control group (*p*<0.05). We observed 15.54%, 20.86%, and 41.61% positivity rates on the third, fifth, and seventh days of incubation, respectively (Figure 3B).

### Impact of *Btk* on fumonisin B_1_ and B_2_ production by *Fv*

The *Fv* strain produced lower levels of the fumonisins FB_1_ and FB_2_ during interactions with *Btk* LFB-FIOCRUZ (CCGB) 257 for 20 days. In the control group, the average production of FB_1_ and FB_2_ was 18.97 µg/g [relative standard deviation (RSD), 2.2%] and 0.86 µg/g (RSD, 1.2%), respectively. In the treated group, the average production of FB_1_ was 13.81 µg/g (RSD, 5.9%), while FB_2_ was not detected in any replicate. The five replicates of the control and treated groups were significantly different from one another (*p*<0.05).

## Discussion

The aim of this study was to characterize the interaction between *Fv* and *Btk* based on the combined use of microscopy, flow cytometry, and HPLC techniques. We also presented a practical and effective approach to identify these interactions using flow cytometry, which could be extended to other plant pathogens and antagonistic bacteria, thereby contributing to the ability to determine the potential of microorganisms as possible BCAs.

Flow cytometry has emerged as a high-resolution technology that supports the characterization of individual cell types within mixed populations. Cellular patterns can be identified by assessing the protein expression using fluorescent probes and antibodies coupled with fluorochromes [39]. This method provides an analysis of a large number of cells and can identify changes within a population and between different populations [33]. Consequently, it was possible to characterize *Fv* in terms of living and dead cell percentages and identify the Cry 1Ab protein produced by *Btk* during the experimental period.

Interaction studies of two or more microorganisms are challenging because the populations are always changing over time. In general, fungal and bacterial cells can grow and divide, leading to an increase in their biomass. *B. thuringiensis* forms a parasporal crystal during the stationary phase of its growth cycle; nevertheless, the co-culture can change the kinetics of both microorganisms, imposing the need to mark each population to monitor their outcome during the interaction.

The fungal cells were labeled with the fluorochrome CFW that binds to chitin, a cell-wall polysaccharide that maintains the fungal cell integrity and confers structural rigidity during growth and morphogenesis [40]. The concentration of CFW was determined based on the background fluorescence, as high concentration levels could be misinterpreted as a positive signal. The optimal concentration was 0.05 µg/mL in 400 µL of the cell solution. We also evaluated the fungal cell autofluorescence by fluorescence microscopy and flow cytometry. The fungal cells showed negligible autofluorescence emission, and therefore, no data normalization was required. Thus, the variation of CFW during the incubation period was interpreted as an increase or decrease of the number of Fv cells. Because CFW has the ability to stain both living and dead cells, the use of the 7-AAD fluorochrome was crucial to determining whether the fungal cells were alive or in an apoptotic stage [39], as the 7-AAD binds only to late apoptotic and necrotic cell DNA. The flow cytometry analysis was not conducted with both markers in the same solution, however, as the CFW could be partially detected in the PerCP channel, which would lead to an overestimation of the 7-AAD positive reactions.

The *Btk* cells were treated with rabbit polyclonal antibody to the *Bt* Cry 1Ab toxin, and a goat polyclonal secondary antibody to rabbit IgG coupled to Alexa Fluor 488, to quantify the Cry 1Ab toxin and determine the bacterial sporulation during the analysis period. Due to the parasporal characteristic of the crystals, which are produced during the stationary phase of *Bt* growth [41], it is possible to make an analogy between the crystal and spore production. The flow cytometry analysis allowed for the characterization of the Cry 1Ab toxin in the *Btk* and *Btk*+*Fv* groups. For both groups, background fluorescence was absent when control cells were treated only with the secondary antibody. This technique enabled the quantification of the Cry 1Ab toxin in terms of percentage. To our knowledge, this is the first report of flow cytometry use for characterizing Cry 1Ab production.

A strong *in vitro* antagonistic effect of the *Btk* LFB-FIOCRUZ (CCGB) 257 strain on *Fv* MRC 826 was shown. Previous studies have reported *Bacillus* spp. as potential biological controls of plant pathogenic fungi such as *F. oxysporum*, *F. sambucinum*, and *F. graminearum* [14], [42]–[44]. In addition, other studies have shown that the activity of *Bacillus* spp. reduced *Fv* colonization and the accumulation of fumonisin in corn [12], [13], [45], [46].

The microscopic examination by SEM and TEM revealed no marked changes on the third day of incubation; however, the antagonistic effect was evident on the fifth and seventh days, showing fungal cell wall and cytoplasm damage in comparison to the control *Fv* culture. Sectors of intumescent hyphae were observed in response to the bacterial effects, with a paucity of fungal growth and numerous *Bt* cells seen in the SEM and indirect immunofluorescence assays. In fact, swollen hyphae can be associated with a decrease in fungal growth and higher levels of non-viable cells due to hyphal transformations and death resulting from *Fusarium* growth kinetics [47]. Another hypothesis is the chitin apposition in the fungal cell wall, due to the stress caused by the effect of antagonistic bacteria. The extensive accumulation of chitin is part of an intricate defense strategy by fungi for restraining the penetration of pathogens and fungitoxic molecules [48], [49]. Nonetheless, it has been reported that *Bacillus* spp. are able to overcome such barriers and cause severe fungal cell injuries [11]. The cell wall and cytoplasm damage visualized by TEM and the profile of dead fungal cells observed by flow cytometry after incubating *Fv* MRC 826 and *Btk* LFB-FIOCRUZ (CCGB) 257 together for 7 days demonstrated that *Bt* strains can be very aggressive towards fungi and are able to circumvent this fungal defense strategy.

Fungal endophytes such as *Fusarium* can reside in the internal tissues of living plants without causing any immediate or negative effect, but may turn pathogenic during host senescence. The interactions between host plants and endophytes in natural populations are poorly understood; however, they can actively compete against other microorganisms for niche or infection sites [50]. *Fv* is an endophyte and one of the most commonly reported soil-borne fungal pathogens infecting maize worldwide [12], [51]. In general, soil-borne pathogens that infect through mycelial contact are more susceptible to competition from other soil microorganisms associated with plants than from pathogens that germinate directly on the plant surface [14], [50]. Our *in vitro* results corroborate this theory, as *Fv* MRC 826 growth was restricted by *Btk* LFB-FIOCRUZ (CCGB) 257 for over 20 days. A reduction in the cell numbers in the treated cultures was also observed on the third (10.93%), fifth (4.19%), and seventh (3.48%) days through the flow cytometry analysis.

The suppressive effect of *Btk* on *Fv* can also be correlated with secreted inhibitory molecules. A number of antifungal compounds, including polypeptides that interact with the fungal membrane, are produced by *Bacillus* species [52]. *Bt* can secrete chitinases and chitin-binding proteins; together these proteins control fungi by binding to cell wall chitin and disrupting the cell polarity, leading to fungal growth inhibition, and thus conferring an advantage as a competitor in its niche. Degraded chitin can also serve as a nutrient and further contribute to *Bt* growth and proliferation [20], [53], [54]. Interestingly, it has been shown that a chitin-binding protein is expressed in spore mother cells that interact with Cry 1Ac, potentiating the toxin activity [20].

Our results showed increased levels of the Cry 1Ab toxin when *Btk* was interacting with *Fv*. The accumulation of Cry 1ab was enhanced during the interaction, particularly after 7 days of incubation. This response probably occurred as a consequence of the stressful conditions during the *Fv* and *Btk* co-cultivation, which may promote more aggressiveness in this *Btk* isolate. Given that *Bt* crystals are produced concurrently with the spores, there was also an increase in spore production during the experimental period. Despite the controversial hypotheses about *Bt* spore longevity, some reports have demonstrated the environmental persistence of *Bt* spores, which confer resistance and a competitive advantage against other microorganisms [19], [27].

During the *Fv* and *Btk* interactions, less accumulation of fumonisin was observed. Reduced FB_1_ levels have been reported previously, when *Fv* was treated with *Bacillus* spp. in maize [12], [13]. In fact, reduced fumonisin levels were reported when the fungal biomass was decreased [13], [55], [56]. Mycotoxin production has been viewed as an adaptation of the fungus to stressful environmental conditions and as providing an advantage in competition with other microorganisms in its natural habitat [57]. Some stressful conditions such as oxidative stress and nitrogen starvation may enhance fumonisin production. However, the benefit to the fungus from producing higher levels of fumonisin remains unclear [58], [59].

In conclusion, our findings are important for agricultural applications since *Btk* LFB-FIOCRUZ (CCGB) 257 exhibited strong biocontrol potential for suppressing *Fv* growth and inhibiting fumonisin production *in vitro*. Moreover, flow cytometry was demonstrated to be sensitive enough to characterize fungal cell oscillations and death during its interaction with *Btk*. Future studies regarding the antagonistic effect of this bacterium against other fungi and *in vivo* tests are necessary to determine the efficacy of *Btk* in controlling plant pathogens.
