# Correction: Multi-Method Approach for Characterizing the Interaction between *Fusarium verticillioides* and *Bacillus thuringiensis* Subsp. *Kurstaki*

**DOI:** 10.1371/journal.pone.0105840

**Published:** 2014-08-12

**Authors:** The PLOS ONE Staff

Figures 2 is missing portions 2b3, 2b4, 2c1, 2c2 and 2c3. Please see the complete, correct Figure 2 here.

**Figure 2:**
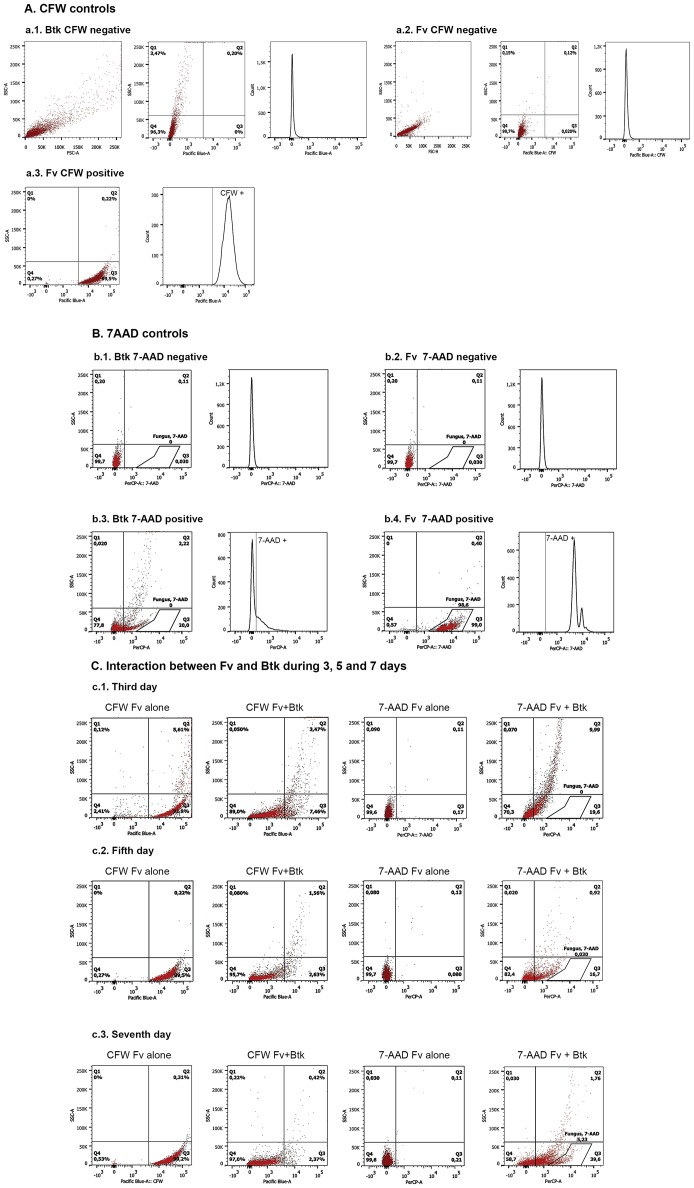
Flow cytometry analysis for *Bacillus thuringiensis* subsp. *kurstaki* (*Btk*) interacting with *Fusarium verticillioides* (*Fv*). Histograms show the number of cells versus the fluorescence intensity. Dot plot graphs show the cell size (SSC) versus the cellular complexity and the SSC versus the fluorescence intensity. The vertical lines define the baseline above which the fluorescence is positive. Figure 2(A). Calcofluor White (CFW) controls and their respective histograms: negative, *Btk* stained with CFW (a.1); negative, *Fv* cells not stained with CFW (a.2); and positive, fungal cells stained with CFW (a.3). )Figure 2(B. 7-Aminoactinomycin (7-AAD) controls: negative, living *Btk* cells (b.1.); negative, living *Fv* cells (b.2.); positive, dead *Btk* cells (b.3.); and positive, dead *Fv* cells (b.4.). The fungal cells were gated based on the forward (FSC) and side scatter (SSC) and previous analyses with 7AAD. Figure 2(C). Analysis of the *Fv* and *Fv*+*Btk* cells labeled with CFW and 7-AAD after 3 (c.1), 5 (c.2), and 7 (c.3) days.
