# Correction: Correction: Multi-Method Approach for Characterizing the Interaction between *Fusarium verticillioides* and *Bacillus thuringiensis* Subsp. *Kurstaki*

**DOI:** 10.1371/journal.pone.0141522

**Published:** 2015-10-21

**Authors:** Liliana O. Rocha, Sabina Moser Tralamazza, Gabriela M. Reis, Leon Rabinovitch, Cynara B. Barbosa, Benedito Corrêa

Fig 4 is missing portion 4b. Please see the complete, correct Fig 4 here. This correction is in addition to the correction for Fig 2.

**Fig 4:**
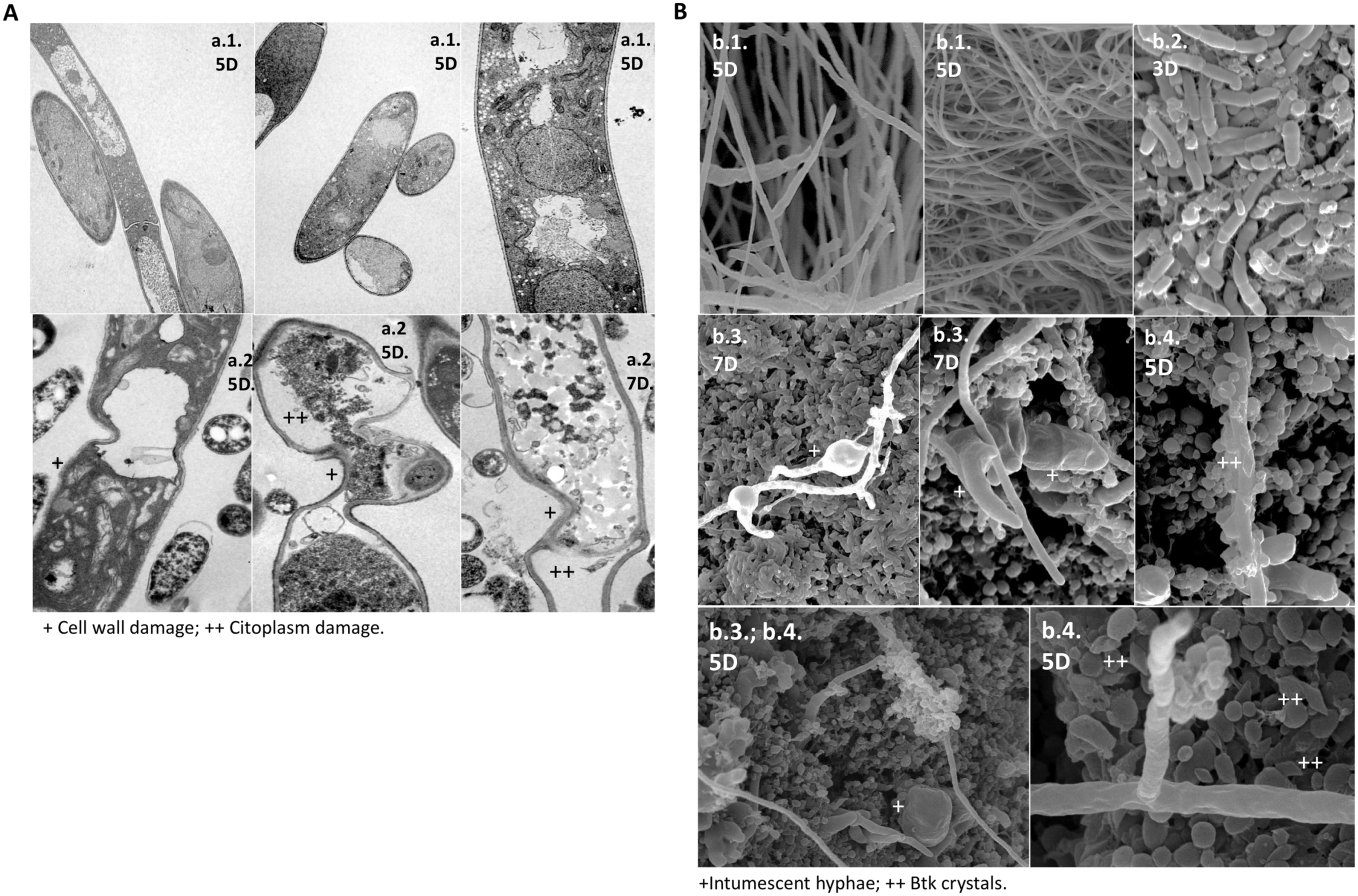
Microscopic observations of *Fusarium verticillioides* (Fv) in contact with *Bacillus thuringiensis* subsp. *kurstaki* (*Btk*). *Fv* and *Btk* were co-cultured on potato dextrose agar and microscopically examined after 5 (5D) and 7 (7D) days of growth. **Figure 4(A)**. Transmission electron microscopy (TEM): *Fv* cells from pure culture (a.1) and with damaged cell walls and disorganization of the cytoplasm—scale bar: 1 μm (a.2). **Figure 4(B)**. Scanning electron microscopy (SEM): *Fv* cells from pure culture—scale bars: 10 μm and 20 μm (b.1); *Btk* cells from pure culture—scale bar: 10 μm (b.2); intumescent hyphae and sparse fungal growth—scale bars: 5 μm, 10 μm and 20 μm (b.3); and *Btk* spores and crystals around the hyphae—scale bars: 5 μm and 10 μm (b.4).
