# Correction: Multi-Method Approach for Characterizing the Interaction between *Fusarium verticillioides* and *Bacillus thuringiensis* Subsp. *Kurstaki*

**DOI:** 10.1371/journal.pone.0216693

**Published:** 2019-05-09

**Authors:** Liliana O. Rocha, Sabina Moser. Tralamazza, Gabriela M. Reis, Leon Rabinovitch, Cynara B. Barbosa, Benedito Corrêa

Following publication of this article [1] and the posting of Corrections to Figures 2 and 4 [2, 3], concerns were raised about duplicate flow cytometry dot plots in Figs 2 and 3.

There is an error in Fig 2 in both the original article and the previous correction [1,2]. Specifically, the b.1.“*Btk* 7-AAD negative” panel is a duplicate of the b.2. “*Fv* 7-AAD negative” panel. Here a revised Fig 2 is provided, with the b.1. “*Btk* 7-AAD negative” panel replaced with the correct plot from the original experiment.

**Fig 2:**
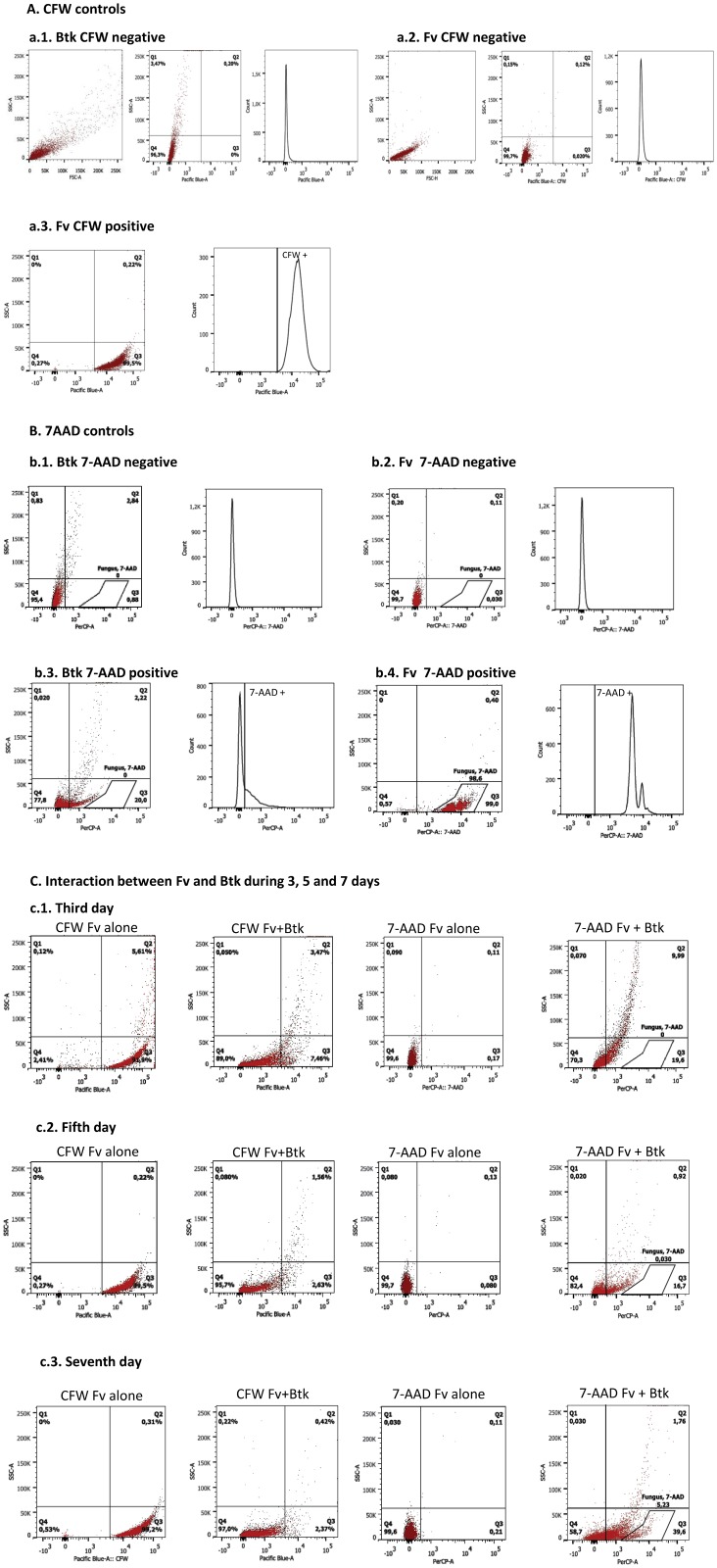
Flow cytometry analysis for Bacillus thuringiensis subsp. kurstaki (Btk) interacting with Fusarium verticillioides (Fv). Histograms show the number of cells versus the fluorescence intensity. Dot plot graphs show the cell size (SSC) versus the cellular complexity and the SSC versus the fluorescence intensity. The vertical lines define the baseline above which the fluorescence is positive. **Figure 2(A)**. Calcofluor White (CFW) controls and their respective histograms: negative, *Btk* stained with CFW (a.1); negative, *Fv* cells not stained with CFW (a.2); and positive, fungal cells stained with CFW (a.3). **Figure 2(B)**. 7-Aminoactinomycin (7-AAD) controls: negative, living *Btk* cells (b.1.); negative, living *Fv* cells (b.2.); positive, dead *Btk* cells (b.3.); and positive, dead *Fv* cells (b.4.). The fungal cells were gated based on the forward (FSC) and side scatter (SSC) and previous analyses with 7AAD. **Figure 2(C)**. Analysis of the *Fv* and *Fv*+*Btk* cells labeled with CFW and 7-AAD after 3 (c.1), 5 (c.2), and 7 (c.3) days.

Additionally, in Fig 3, the a.3. “*Btk* cry 1Ab positive” and b.2. “Fifth day *Btk* alone” panels are duplicates because they represent the same sample. There is an error in the legend of Fig 3, which incorrectly states that the a.3. panel shows a mixed *Btk*+*Fv* cell suspension. The a.3. panel shows day 5 *Btk* cells alone treated with anti-cry 1Ab toxin and secondary antibodies. A revised Fig 3 is provided with accompanying revised figure legend as follows:

**Fig 3:**
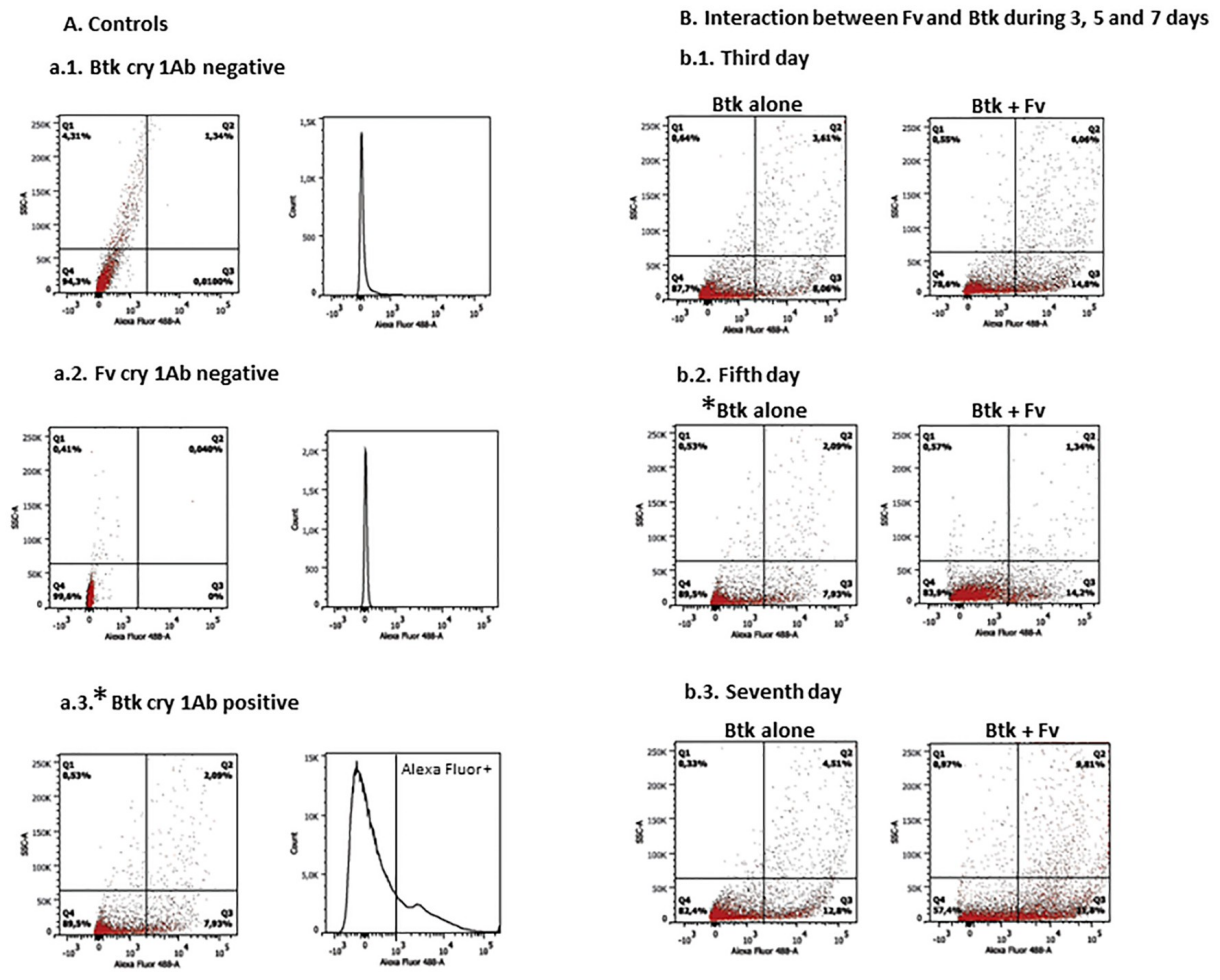
Flow cytometry analysis for quantification of Cry 1Ab toxins. Histograms show the number of Cry 1Ab toxins against the fluorescence intensity. Dot plot graphs show protein size (SSC) against fluorescence intensity. The vertical lines define the baseline above which the fluorescence is positive. (A) Experimental controls. (a.1.) Negative control–*Btk* cell suspension treated with secondary antibody coupled with Alexa Fluor 488; (a.2.) Fv cells treated with the first and secondary antibodies; (a.3.) Positive control–*Btk* cell suspension treated with antibody to Cry 1Ab toxin and secondary antibody. (B) Analysis of Cry 1Ab production during 3, 5 and 7 days of *Fv* + *Btk* interaction. (b.1.) Third day: *Btk* + *Fv* interaction and *Btk* Cry 1Ab toxin stained with Alexa Fluor 488; (b.2.) fifth day; (b.3.) seventh day. * Fifth day, *Btk* alone was also used as positive control for *Btk*, with the purpose of showing the positive histogram for Cry 1Ab marked with Alexa Fluor 488.

Flow cytometry analysis output files for the above panels are provided as Supporting Information files below. Additionally, the raw .fcs files for the above panels and additional replicates are provided as Supporting Information. However, the raw flow cytometry data for the other panels in Figs 2 and 3 and the underlying data files for the other figures are no longer available.

The previous Correction to Figure 4 [3] continues to apply.

## Supporting information

S1 File Fig 2 Data. (ZIP)

S2 File Fig 3 Data. (ZIP)
