# Supplementary figures and images for: Correction: Multi-Method Approach for Characterizing the Interaction between Fusarium verticillioides and Bacillus thuringiensis Subsp. Kurstaki
Source: PLoS One. 2019 May 9;14(5):e0216693. doi: 10.1371/journal.pone.0216693 (PMC6508605; doi:10.1371/journal.pone.0216693)

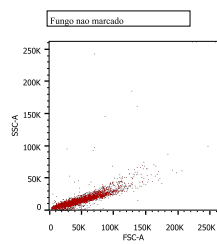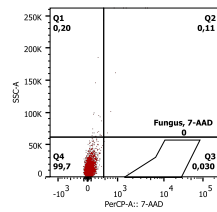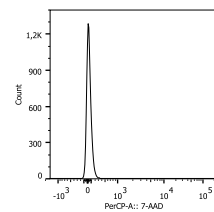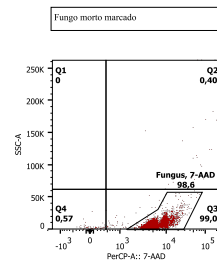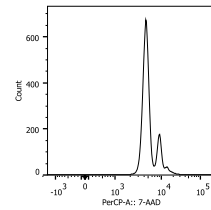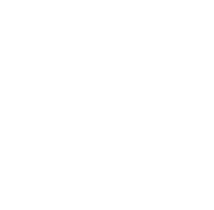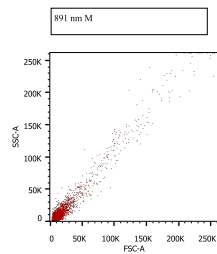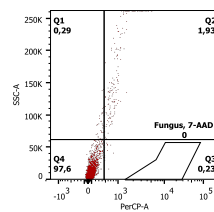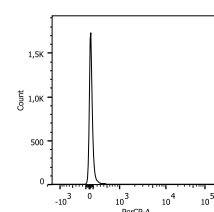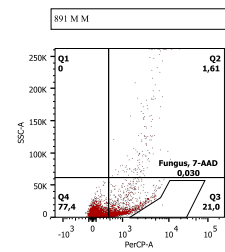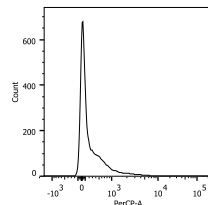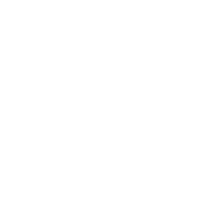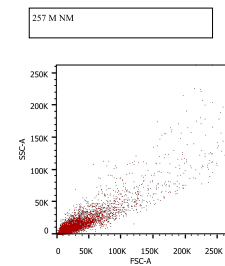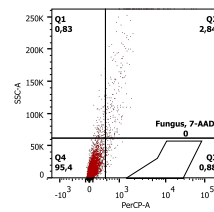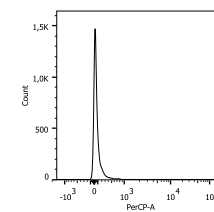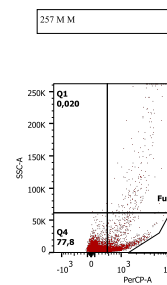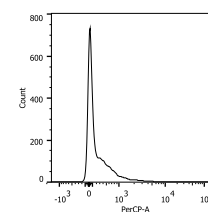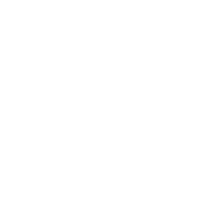

Supplement: S1 File — (ZIP) [file pone.0216693.s001.zip › S1 File/7AAD_controls flowjoutput.pdf]

Fgo nm

Fgo marcado com AC

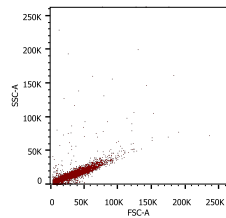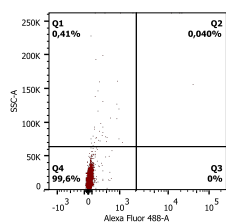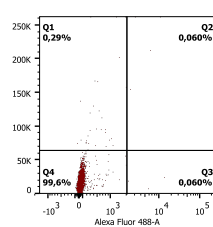

SSC-A

257 nm

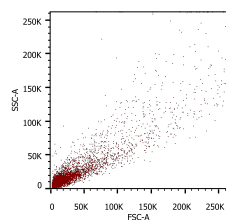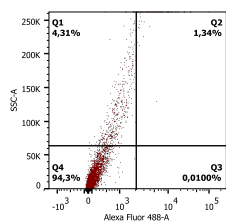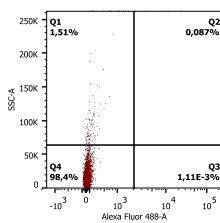

257 3d + AC

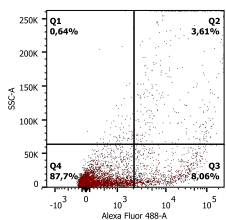

257 5d + AC

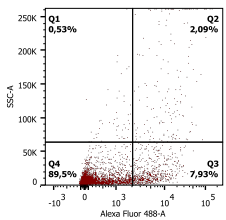

257 7d + AC

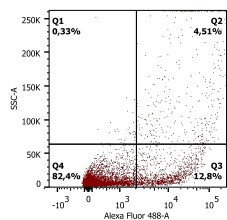

Supplement: S2 File — (ZIP) [file pone.0216693.s002.zip › S2 File/cry 1Ab controls no histogram flowjoutput (1).pdf]

Fgo m

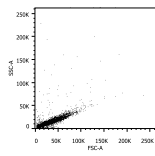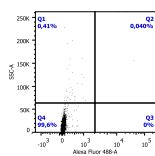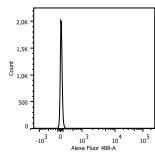

Fgo marcado com AC

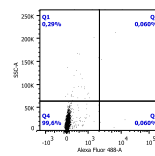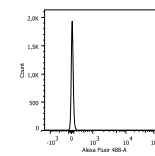

257 m

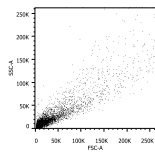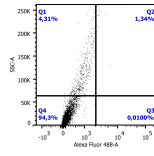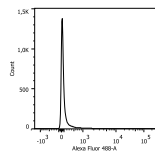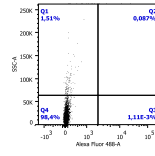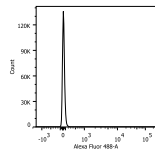

257 M + AC

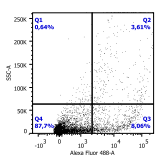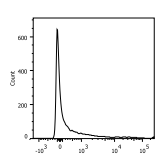

257 S4 + AC

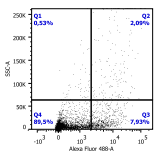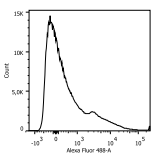

257 T6 + AC

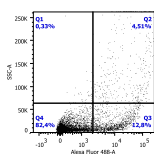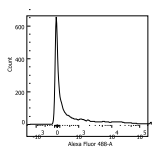

257 + F M + AC

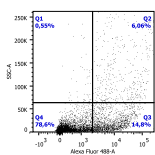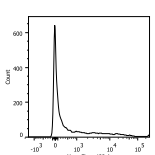

257 + F S4 + AC

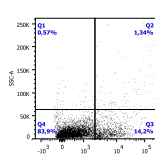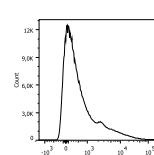

257 + F M + AC

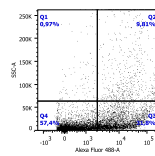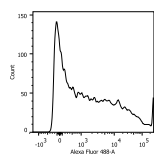

Supplement: S2 File — (ZIP) [file pone.0216693.s002.zip › S2 File/cry 1Ab_control_andFvdays flowjoutput (1).pdf]
